# 2-Amino metabolites are key mediators of CB 1954 and SN 23862 bystander effects in nitroreductase GDEPT

**DOI:** 10.1038/sj.bjc.6601612

**Published:** 2004-03-02

**Authors:** N A Helsby, D M Ferry, A V Patterson, S M Pullen, W R Wilson

**Affiliations:** 1 Auckland Cancer Society Research Centre, Faculty of Medical and Health Sciences, University of Auckland, Private Bag 92019, Auckland, New Zealand

**Keywords:** CB 1954, SN 23862, prodrugs, bystander effect, GDEPT, nitroreductase

## Abstract

An important feature of gene-directed enzyme-prodrug therapy is that prodrug activation can provide diffusible cytotoxic metabolites capable of generating a local bystander effect in tumours. Activation of the aziridinyl dinitrobenzamide CB 1954 by *E. coli* nitroreductase (NTR) provides a bystander effect assumed to be due to the potently cytotoxic 4-hydroxylamine metabolite. We show that there are four cytotoxic extracellular metabolites of CB 1954 in cultures of NTR-expressing tumour cells (the 2- and 4-hydroxylamines and their corresponding amines). The 4-hydroxylamine is the most cytotoxic in DNA crosslink repair defective cells, but the 2-amino derivative (CB 10-236) is of similar potency to the 4-hydroxylamine in human tumour cell lines. Importantly, CB 10-236 has much superior diffusion properties to the 4-hydroxylamine in multicellular layers grown from the SiHa human cervical carcinoma cell line. These results suggest that the 2-amine, not the 4-hydroxylamine, is the major bystander metabolite when CB 1954 is activated by NTR in tumours. The corresponding dinitrobenzamide nitrogen mustard SN 23862 is reduced by NTR to form a single extracellular metabolite (also the 2-amine), which has superior cytotoxic potency and diffusion properties to the CB 1954 metabolites. These results are consistent with the reported high bystander efficiency of SN 23862 as an NTR prodrug in multicellular layers and tumour xenografts.

In gene-dependent enzyme-prodrug therapy (GDEPT), gene therapy methods are used to express a prodrug-activating enzyme in tumours. An important aspect of this approach is its ability to generate cytotoxic metabolites that diffuse locally to cause a bystander effect, thereby compensating in part for spatially inhomogeneous transgene expression. Activation of the dinitrobenzamide aziridine prodrug CB 1954 (Figure 1

**Figure 1:**
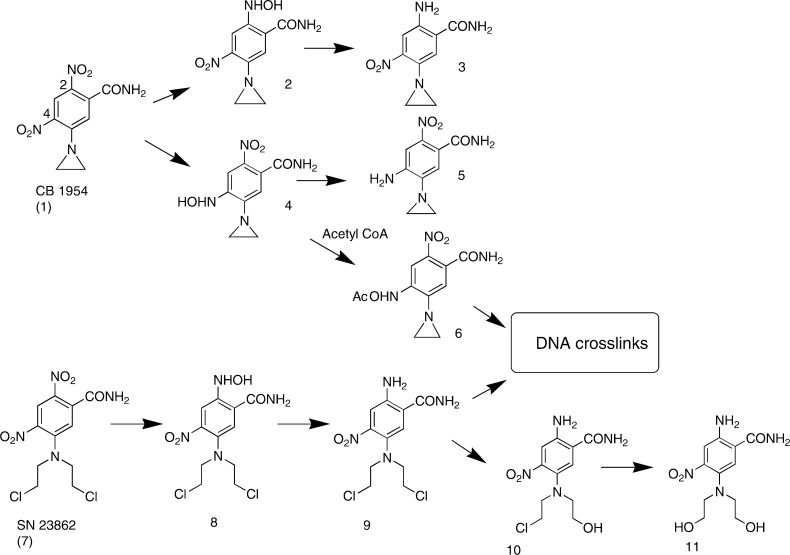
Products of the metabolism of the dinitrobenzamide prodrugs CB 1954 and SN 23862 by *E. coli* nitroreductase.

) by the aerobic *E. coli* nitroreductase NTR (the product of the *NfsB* gene) has attracted considerable attention in this context, because it provides an efficient bystander effect in tissue culture models (Bridgewater *et al*, 1997; Green *et al*, 1997; Friedlos *et al*, 1998; McNeish *et al*, 1998; Nishihara *et al*, 1998; Westphal *et al*, 2000; Wilson *et al*, 2002) and in experimental tumours (McNeish *et al*, 1998; Djeha *et al*, 2000; Westphal *et al*, 2000; Wilson *et al*, 2002). The combination of CB 1954 with NTR-armed adenoviral vectors is currently in clinical trial for cancer therapy (Chung-Faye *et al*, 2001; Young *et al*, 2002). It is widely considered that the active cytotoxic metabolite from CB 1954 (whether reduced by NTR, DT-diaphorase or other reductases) is the corresponding 4-hydroxylamine (**4** in Figure 1), and that this is the species responsible for bystander effects in NTR-GDEPT. Reduction of CB 1954 with NTR plus NADH in cell-free systems gives an equimolar mixture of the 2- and 4-hydroxylamines (Boland *et al*, 1991; Anlezark *et al*, 1992) (Figure 1), and the 4-hydroxylamine has been shown to be the more cytotoxic of the two metabolites (Knox *et al*, 1988b; Sunters *et al*, 1992; Helsby *et al*, 2003). There is considerable evidence that the potent cytotoxicity of the 4-hydroxylamine is related to its ability to form DNA crosslinks via the *N*-acetoxy intermediate **6** (Knox *et al*, 1991).

The evidence that the 4-hydroxylamine of CB 1954 is responsible for its bystander effects rests largely on a study which tentatively identified the 2- and 4-hydroxylamines in the extracellular medium, following incubation of NTR-expressing 3T3 cells with CB 1954 (Bridgewater *et al*, 1997). It has been reported previously that metabolism of CB 1954 by mouse liver microsomes gives the 2- and 4-amines as the end products (Walton *et al*, 1989), and the amines (predominantly the 4-amine) rather than hydroxylamines are identified as metabolites in rodents (Jarman *et al*, 1976; Kestell *et al*, 2000). In addition, the hydroxylamine derivatives of CB 1954 are chemically unstable (Knox *et al*, 1993; Helsby *et al*, 2003), which makes it unclear whether they will be able to diffuse appreciable distances in tumour tissue. These considerations have led us to revisit the identification of the metabolite(s) that mediates the CB 1954 bystander effect. In this study, we characterise extracellular metabolites in CB 1954-treated NTR+ve cell cultures and investigate their extravascular transport properties in multicellular layer (MCL) cultures. The latter are three-dimensional cell cultures useful for quantifying the tissue-diffusion properties of drugs and metabolites (Hicks *et al*, 2001,^2003^; Wilson *et al*, 2003).

In addition, we evaluate bystander metabolites from the corresponding nitrogen mustard analogue SN 23862 (Palmer *et al*, 1992). This has been suggested as an alternative NTR prodrug because of its faster kinetics of reduction by NTR (Anlezark *et al*, 1995) and more efficient bystander effect in MCL cultures and human tumour xenografts (Wilson *et al*, 2002). Although the 4-NO_2_ group is the thermodynamically preferred site of reduction (Palmer *et al*, 1995), SN 23862 is reduced by NTR at the 2-NO_2_ position only (Figure 1) (Anlezark *et al*, 1995); this increases the reactivity of the nitrogen mustard moiety 260-fold, and thus provides a single-step bioactivation of the prodrug, without any requirement for *N*-acetoxy formation (Helsby *et al*, 2003).

## MATERIALS AND METHODS

### Chemicals

CB 1954 (**1**) was supplied by Professor M Jarman (Institute for Cancer Research, Sutton, UK), and its amino derivatives 3 and 5 by Professor Richard Knox (Enact Pharma plc, Porton Down Science Park, Salisbury, Wiltshire SP4 0JQ, UK). The 2-and 4-hydroxylamines of CB 1954 (**2** and **4**) (Jarman *et al*, 1976), SN 23862 (7) (Palmer *et al*, 1992) and its 2-hydroxylamine and 2-amine derivatives **8** and **9** (Palmer *et al*, 1995) were synthesised as reported. All compounds showed purities of >98% by HPLC, with the exception of **2**, which contained up to 4% of **3** and 2% of the 2,2′-azoxy dimer, and **8**, which contained up to 20% of **9**.

### Cell lines

The V79-NTR^*puro*^ line (originally named T79-A3) is a stable NTR transfectant and V79^*puro*^ (originally T78-1) is an NTR−ve clone transfected with the corresponding empty vector (Friedlos *et al*, 1997). A second NTR-expressing line, SiHa-NTR^*puro*^, was developed by transfecting the SiHa human cervical carcinoma line with plasmid F399, a derivative of pEFIRES-P (Hobbs *et al*, 1998), containing a bicistronic cassette in which the NTR and *pac* genes are co-transcribed from a single EF-1*α* promoter. The pool was selected in increasing concentrations of puromycin (up to 3 *μ*M). An isolated clonogen, SiHa-NTR^*puro*^, had growth rates similar to SiHa and retained CB 1954 sensitivity (440-fold relative to the parental line in 4-h exposure IC_50_ assays) during growth for 4 weeks in the absence of puromycin selection. Cell lines were passaged as monolayers in *α*MEM containing 5% foetal bovine serum (FBS), with added puromycin (15 *μ*M for V79-NTR^*puro*^, 3 *μ*M for SiHa-NTR^*puro*^) in the case of the NTR-expressing lines, for up to 12 weeks from frozen stocks that were confirmed to be free of mycoplasma by PCR-ELISA (Roche Diagnostics).

### Prodrug metabolism in single-cell suspensions

Cells were trypsinised from late log-phase monolayers and resuspended in *α*MEM containing added ascorbate (0.25 mM) with or without 5% FBS to densities up to 5 × 10^6^ cells ml^−1^. The single-cell suspensions (10 ml) were stirred magnetically and gassed continuously with 5% CO_2_/air in a 37°C water bath. Following equilibration for 1 h, CB 1954 and SN 23862 were added from DMSO stock solutions to give concentrations up to 100 *μ*M (final DMSO concentration ⩽0.5%). At various times, samples were centrifuged to pellet cells and the extracellular medium was freshly supplemented with ascorbate (1 mM) and stored at −80°C for subsequent HPLC analysis.

### HPLC, mass spectrometry and fraction collection

In experiments without FBS, samples of extracellular medium were thawed individually, centrifuged (10 000 *g* × 2 min), and analysed immediately (within 3 min) by HPLC, without further processing. For samples containing FBS, 2 v methanol was added and the samples were held at −20°C to precipitate proteins before centrifugation, concentrated to ca 170 *μ*l in a centrifugal concentrator (Savant Instruments, Farmingdale, NY, USA), centrifuged again and assayed immediately. The HPLC system was an Agilent 1100 (Agilent, Waldbronn, Germany) with an Alltech Altima 3.2 × 150 mm C8 5*μ* column (Alltech Associates Inc., Deerfield, IL, USA). The mobile phase for the bioassay experiments comprised linear gradients of acetonitrile in water (4–56% acetonitrile for 2–15 min, decreasing to 4% at 17 min for CB1954; 16–64% acetonitrile for 2–6 min, decreasing to 16% between 12 and 15 min for SN 23862) at a flow rate of 0.5 ml min^−1^. In all other experiments, 0.45 M ammonium formate buffer (pH 6.5 for CB 1954, pH 4.5 for SN 23862) replaced the water in the mobile phase. Detection was by diode array absorbance at 330 nm for CB 1954 and 272 nm for SN 23862 (bandwidth 4 nm, reference wavelength 500–600 nm). Phenol red in the culture medium was used as internal standard to correct for the evaporative concentration of samples during incubation. In some experiments, metabolites were identified by on-line mass spectrometry using a single-stage quadrupole mass detector (Agilent MSD model D) with negative mode atmospheric pressure chemical ionisation, using N_2_ as the nebulising and drying gas at a flow rate of 5 l min^−1^ and a nebulising pressure of 55 psi. The gas temperature was 350°C, the vaporiser temperature was 450°C, the capillary voltage was 4000 V and the corona current was 4 *μ*A. Mass spectra were also collected using flow-injection analysis of fractions of the HPLC eluate, using an injection cycle time of 0.8 min. HPLC fractions were collected (12-s intervals between 5 and 15 min, and 30-s intervals between 0–5 and 5–20 min) for bioassay and/or off-line mass spectrometry.

### Cytotoxicity assays

The bioactivity of HPLC fractions was determined by immediately diluting 10 *μ*l into cultures of UV4 cells in 96-well plates (seeded at 300 cells in 100 *μ*l per well 24 h previously), and then making three-fold dilutions across the plate. The cultures were incubated in *α*MEM containing 5% FBS at 37°C for 96 h and cell densities determined by staining with sulphorhodamine B (Skehan *et al*, 1990). The cytotoxic potencies of DMSO stock solutions of the prodrugs and synthetic metabolites were determined similarly, but with 4 h drug exposure of Chinese hamster fibroblasts (UV4, V79, AA8) and human tumour cells (SiHa, WiDr), essentially as previously (Wilson *et al*, 1996). IC_50_ values were determined by logistic interpolation as the drug concentration required to reduce cell density to 50% of that of controls on the same plate.

### Transport of potential bystander metabolites through multicellular layers

MCL were grown by seeding 0.6 × 10^6^ SiHa or 1 × 10^6^ SiHa-NTR^*puro*^ cells onto collagen-coated Millicell-CM membranes (Millipore, Bedford, MA, USA) and grown for 4 days, submerged in a stirred chamber of *α*MEM containing 10% FBS, 100 IU ml^−1^ penicillin and 100 *μ*g ml^−1^ streptomycin for MCLs approximately 200 *μ*m in thickness; full details of the method for growing MCLs are given elsewhere (Wilson *et al*, 2003). The MCLs were mounted in a two-chamber diffusion apparatus (Hicks *et al*, 2001) with 8 ml *α*MEM without FBS in each compartment. For SiHa-NTR^*puro*^ MCLs, CB 1954 or SN 23862 was added to the ‘donor’ compartment (100 *μ*M), and the prodrug and metabolites were monitored in both the donor and receiver compartments by storing samples at −80°C, and then directly injecting onto the HPLC within minutes of thawing. Transport of **4** (100 *μ*M initial concentration in the donor compartment) across SiHa^WT^ MCLs was measured similarly.

## RESULTS

### Metabolism of CB 1954 by NTR-expressing cells

The overall rate of CB 1954 metabolism in cells was monitored by measuring the extracellular concentration in stirred cell suspensions, using direct HPLC analysis of the extracellular medium (without FBS). Using NTR+ve cells, disappearance of the prodrug displayed first-order kinetics, and was much faster than with NTR−ve cells (Table 1

**Table 1:** Kinetics of metabolic consumption of dinitrobenzamide prodrugs by NTR-expressing and control cells at 10^6^ cells ml^−1^, determined by monitoring loss with time in the extracellular medium

	**First-order rate constant *k* (h^−1^)**^a^	
**Cell line**	**CB 1954**	**SN 23862**
V79^puro^	0.0010±0.0004	0.0020±0.0001
V79-NTR^*puro*^	0.08±0.03	0.17±0.003
SiHa	0.0066±0.0001	<0.001
SiHa-NTR^*puro*^	0.22±0.06	0.6±0.2

a Experiments were performed using a cell density of 5 × 10^6^ ml^−1^ for the V79 lines and SiHa, and 2.5 × 10^5^ ml^−1^ for SiHa-NTR*^puro^*.

Values are means±s.e.m. for 2–3 cell suspensions, corrected to a cell density of 10^6^ ml^−1^.

). The rate of metabolism by SiHa-NTR^*puro*^ cells was 2.75-fold faster than V79-NTR^*puro*^ cells, consistent with the more intense NTR band observed in Western blots from the former cell line (data not shown).

**Figure 2:**
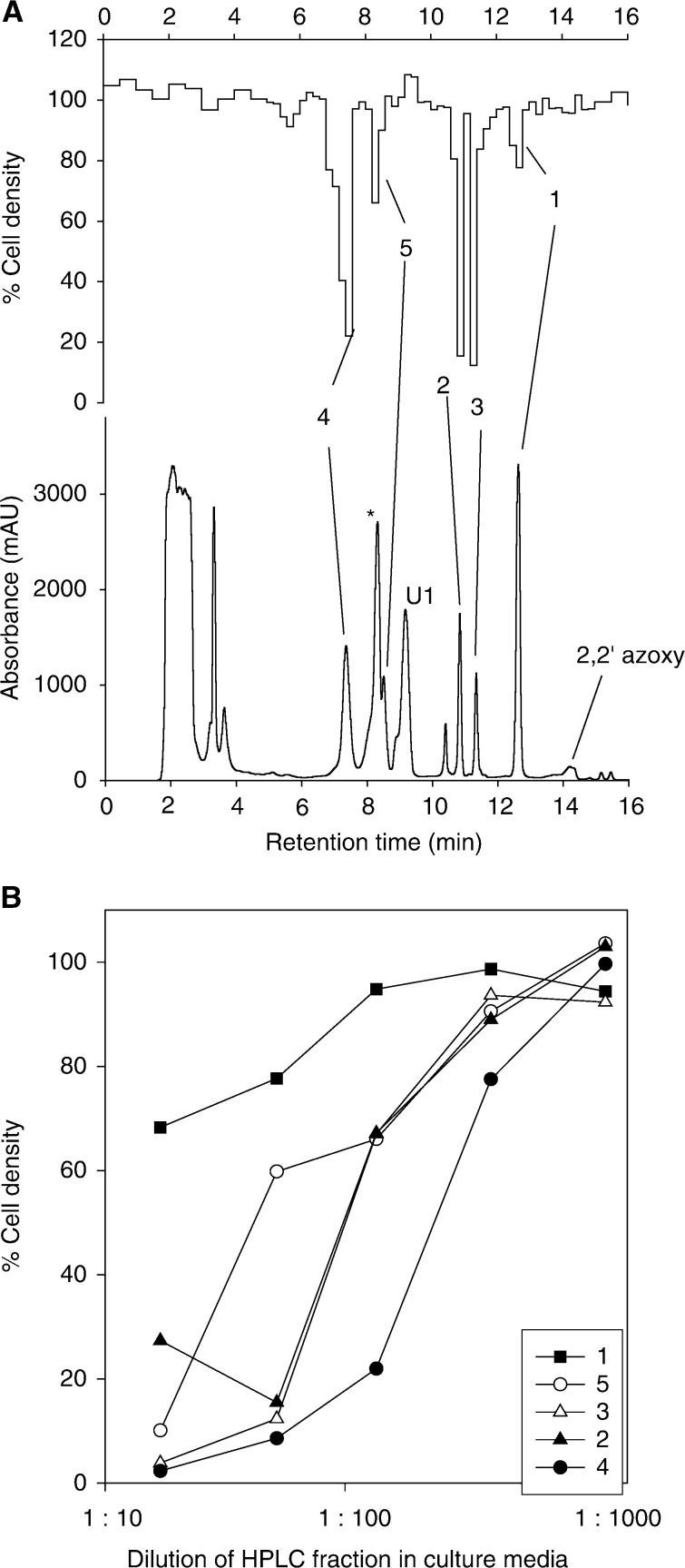
Extracellular metabolites of CB 1954 in V79-NTR^*puro*^ cell suspensions (5 × 10^6^ ml^−1^, 3 h). (**A**) Absorbance chromatogram at 330 nm (lower) and biochromatogram (upper) determined using a 1 : 135 dilution of HPLC fractions into UV4 cell cultures. U1, unknown product; ^*^phenol red. (**B**) Comparison of cytotoxicity of the bioactive extracellular products. The abscissa is the dilution factor in the bioassay.

Consumption of CB 1954 by both NTR+ve cell lines was accompanied by formation of multiple extracellular metabolites, as illustrated by the chromatogram in Figure 2A

(5 × 10^6^ V79-NTR^*puro*^ cells ml^−1^, 3 h). In this experiment, 4 ml of extracellular medium (containing 5% FBS) was deproteinised, concentrated and analysed by HPLC. The metabolites were identified by comparison of retention times, absorbance spectra and on-line mass spectra with authentic (synthetic) standards. The mass spectra gave prominent parent molecular ions at the expected *m*/*z* values: 2-hydroxylamine **2** and 4-hydroxylamine **4** ([M–H]^−^=237.1), 2-amine **3** and 4-amine **5** ([M–H]^−^=221.1) and 2,2′azoxy derivative ([M–H]^−^=455), which is a known product from dimerisation of **2** and the corresponding nitroso compound (Helsby *et al*, 2003). Thus, the major extracellular products from NTR reduction of CB 1954 in V79-NTR^*puro*^ cells include the 2- and 4-amines, as well as the primary hydroxylamine metabolites.

### Bioactivity of CB 1954 metabolites

The eluate from the same chromatogram (Figure 2A) was bioassayed by immediate dilution into cultures of UV4 cells, which are sensitive to alkylating agents because of a defect in nucleotide excision repair (Hoy *et al*, 1985). In addition to the weak cytotoxicity of residual CB 1954, four peaks of bioactivity were detected. The greatest activity co-eluted with the 4-hydroxylamine **4** as expected, but the 2-hydroxylamine (**2**), the 2-amine (**3**) and the 4-amine (**5**) also showed significant cytotoxicity, while the unidentified metabolite U1 and the 2,2′-azoxy dimer were not bioactive. Serial dilution of the HPLC fractions (Figure 2B) confirmed that the most bioactive species was **4**, but the contribution of the other reduction products was only slightly less (and collectively of a similar order to **4**).

This somewhat surprising finding led us to compare the cytotoxic potencies of the synthetic samples of the amine and hydroxylamine reduction products of CB 1954, using IC_50_ assays with 4 h drug exposure and a subsequent growth period of 4 days (Table 2

**Table 2:** Cytotoxicities of the synthetic metabolites of CB 1954 (**1**) and SN 23862 (**7**) against NTR−ve cell lines

	**IC_50_ (*μ*M) following 4 h drug exposure**					
**Cmpd**	**SiHa**	**WiDr**	**V79^puro^**	**AA8**	**UV4**	**AA8/UV4^a^**
**1**	277±36	485±34	2950±113	2480±260	135±28	22.1±5.2
(CB 1954)						
**2**	n.d.^b^	n.d.	n.d.	22	6.3	3.4
**3**	7.9±3.3	21±3	56±7	28±8	3.7±0.7	7.1±1.0
**4**	45±9	26±5	6.9±0.6	39±6	0.62±0.05	63±5
**5**	163±39	141±34	238±6	62±12	47±5	1.2±0.1

**7**	>800	>1000	>500	ca1000^c^	761±89	ca 1.3
(SN 23862)						
**8**	2.07±0.08	1.2±0.1	1.74±0.23	2.3±0.1	0.045±0.001	51±1
**9**	2.5±1.1	2.75±0.15	3.94±0.19	8.8±1.1	0.23±0.04	40±4

Values are mean±s.e.m. for 2–15 (median 4) independent experiments.

a Intra-experiment IC_50_ ratio.

b Not determined.

c IC_50_ above the solubility limit in some experiments.

). This confirmed that **4** is the most potent cytotoxin in the repair-defective UV4 cell line, but unexpectedly was 11–70-fold less potent in the four repair competent cell lines tested. The 2-amine **3** was more toxic than the 4-amine **5**, and was at least as potent as **4** in the human tumour cell lines (SiHa and WiDr). The 4-hydroxylamine and 2-amine both showed higher potency against the ERCC1-deficient line UV4 than its parental line AA8 (63- and seven-fold, respectively), suggesting that both these metabolites form DNA adducts that are substrates for nucleotide excision repair.

### Extravascular diffusion properties of the cytotoxic CB 1954 metabolites

**Figure 3:**
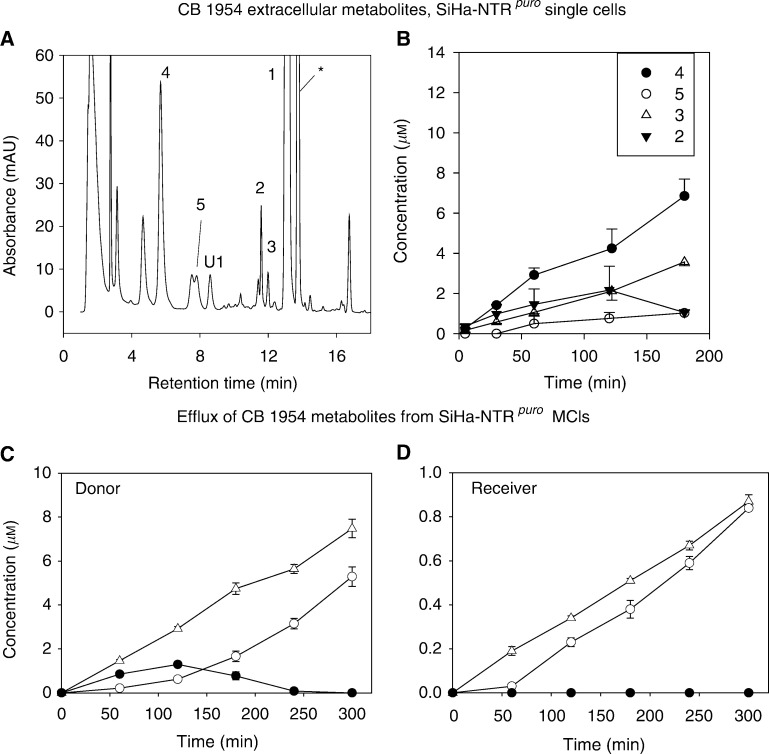
Extracellular metabolites of CB 1954 in SiHa-NTR^*puro*^ single-cell suspensions (**A**, **B**) and multicellular layers (**C**, **D**). (**A**) HPLC chromatogram (absorbance) of extracellular medium after incubation of cells with 100 *μ*M CB 1954 for 3 h at 2.5 × 10^5^ cells ml^−1^. U1, unknown product; ^*^phenol red. (**B**) Time course of formation of extracellular metabolites as in (**A**). (**C**, **D**) Diffusion of CB 1954 metabolites out of SiHa-NTR^*puro*^ MCLs during exposure to CB 1954 (initial concentration 100 *μ*M in the donor compartment). Symbols as in (**B**).

We next compared the ability of all the four cytotoxic metabolites to diffuse in tumour tissue by testing their transport through MCLs. Initially, the metabolites were generated *in situ* in MCLs grown from SiHa-NTR^*puro*^ (Figure 3

); this cell line was used in preference to V79-NTR^*puro*^, since the latter does not grow uniformly as MCLs. It was first demonstrated that SiHa-NTR^*puro*^ gives the same spectrum of extracellular metabolites as V79-NTR^*puro*^ (Figure 3A), and the time course of formation of these metabolites was determined in stirred single-cell suspensions (Figure 3B). This showed that the major extracellular metabolites are the 2- and 4-hydroxylamines and the 2-amine, with lower concentrations of the 4-amine.

To generate the metabolites in SiHa-NTR^*puro*^ MCLs, CB 1954 was added to the donor compartment of the diffusion chamber. As expected, given the CB 1954 concentration gradient through the MCL under these conditions, the metabolites that diffused out of the MCL were at higher concentration on the donor than the receiver side. Notably, the 2- and 4-amines (**3** and **5**) accumulated in both compartments, while the 4-hydroxylamine **4** was observed only transiently at low concentrations in the donor, and was not detected at all in the receiver compartment. The 2-hydroxylamine **2** was not detected in either compartment. Thus, the relative yields of the reduced metabolites in the medium bathing MCLs is quite different from that obtained from single-cell suspensions, and suggests that the relatively stable amine end products have diffusion ranges much greater than the hydroxylamines in tumour tissue.

**Figure 4:**
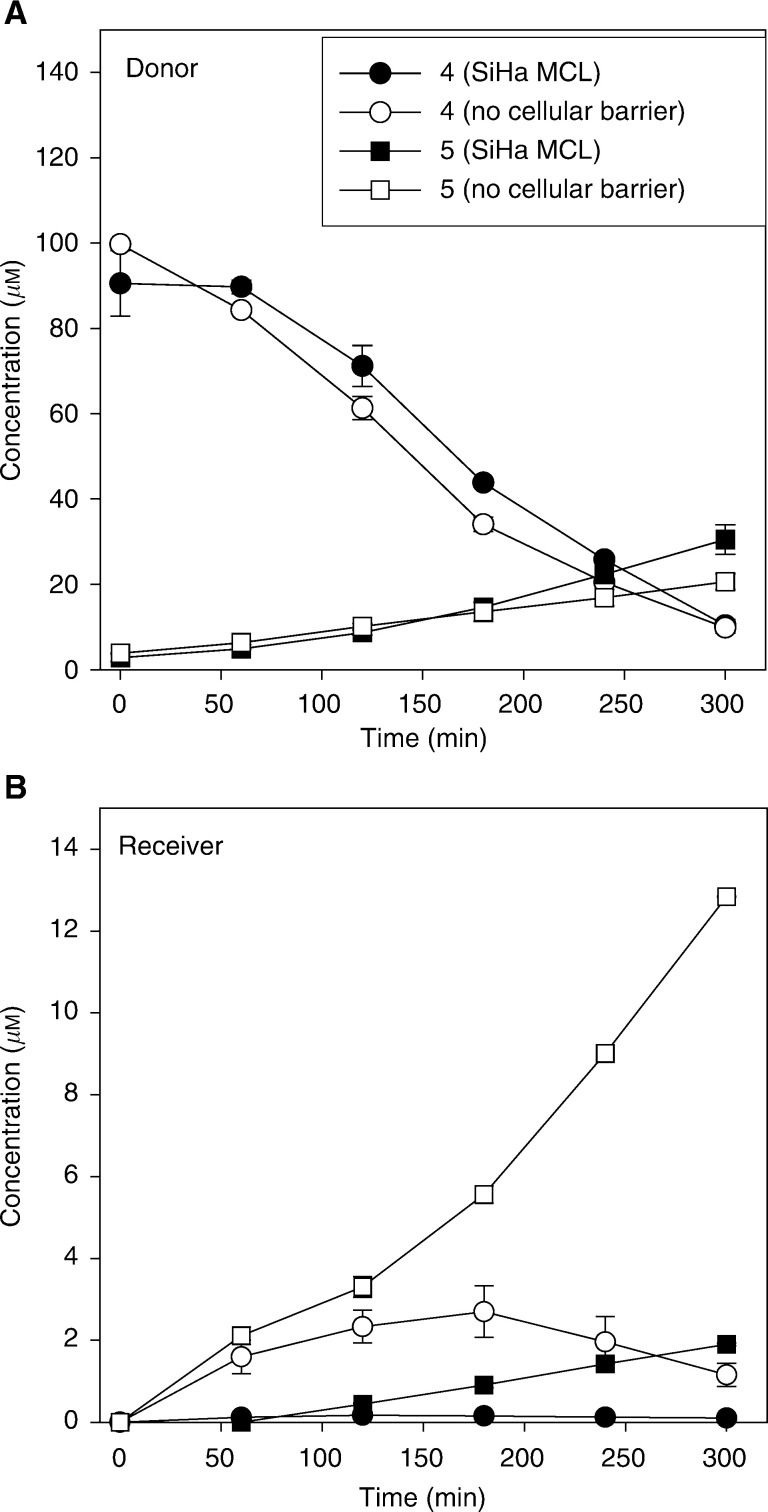
Transport of the 4-hydroxylamine metabolite of CB 1954 (**4**) through bare collagen/Teflon support membranes (open symbols) and through SiHa MCLs (closed symbols), and formation of the corresponding 4-amine (**5**). Values are the mean±range from two MCLs.

To further evaluate the tissue transport properties of the 4-hydroxylamine, **4** (100 *μ*M) was added to the donor compartment of diffusion chambers containing either SiHa MCLs or the bare support membranes (collagen-coated porous Teflon) on which the MCLs are grown (Figure 4

). Rapid loss was observed from the donor in both cases, in part accounted for by the formation of the 4-amine **5**, but the appearance of **4** in the receiver compartment was greatly reduced in the presence of an MCL, confirming its inefficient extravascular diffusion.

### Activation of SN 23862 by NTR-expressing cells and extravascular transport of metabolites

**Figure 5:**
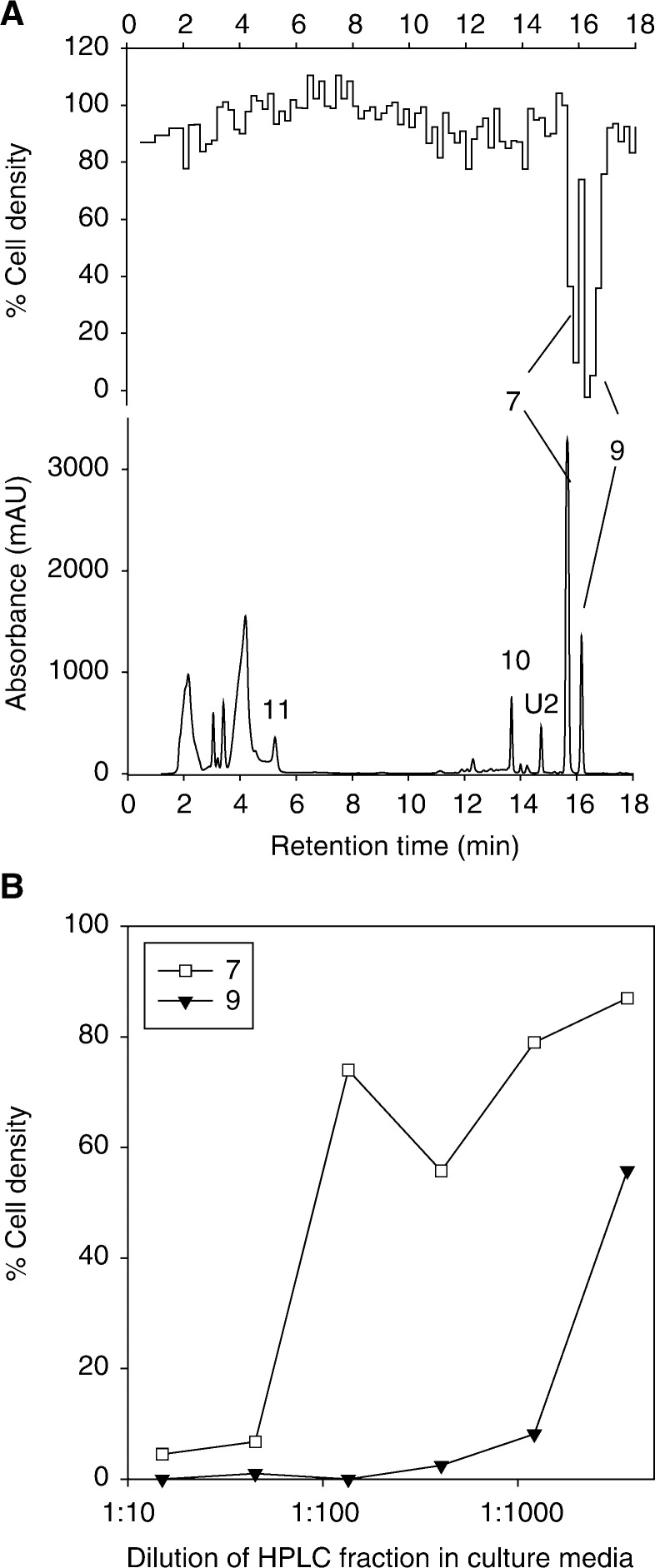
Extracellular metabolites of SN 23862 in V79-NTR^*puro*^ cell suspensions under the same conditions as in Figure 2. (**A**) Absorbance chromatogram at 272 nm (lower) and biochromatogram (upper) determined using a 1 : 135 dilution of HPLC fractions into UV4 cell cultures. (**B**) Comparison of cytotoxicity of the bioactive extracellular products. The abscissa is the dilution factor in the bioassay.

SN 23862 (**7**), a nitrogen mustard analogue of CB 1954, was also consumed by V79-NTR^*puro*^ and SiHa-NTR^*puro*^ cells with first-order kinetics, with higher rate constants than for CB 1954 (Table 1); this is consistent with the improved *k*_cat_ for this substrate (Anlezark *et al*, 1995). Four extracellular metabolites were observed in the medium from V79-NTR^*puro*^ cell suspensions (Figure 5A

); three of these were identified by mass spectrometry and by comparison with synthetic standards as the 2-amine **9** ([M–H]^−^=319) and its corresponding mono-ol (**10**, [M–H]^−^=301) and di-ol (**11**, [M–H]^−^=283) mustard hydrolysis products (see Figure 1). In this case, the 2-amine **9** was the only cytotoxic metabolite detected by bioassay of the HPLC eluate against UV4 cells (Figure 5A upper trace) and, when examined over a range of dilutions, had much greater activity than the residual SN 23862 (Figure 5B). The potent cytotoxicity of **9** was confirmed in IC_50_ assays with authentic compound (Table 2), which demonstrated a similar potency to the 2-hydroxylamine **8** (not detected as an extracellular metabolite) and a higher potency than any of the CB 1954 metabolites in all cell lines (except AA8, in which case **9** and **4** were similar, based on a single determination for **4**).

**Figure 6:**
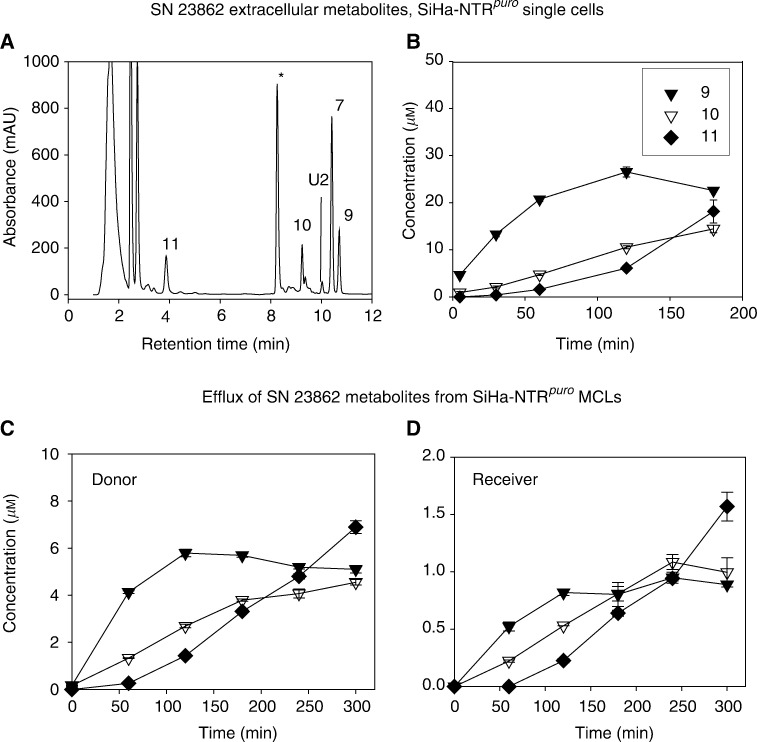
Extracellular metabolites of SN 23862 in SiHa-NTR^*puro*^ single-cell suspensions (**A**, **B**) and multicellular layers (**C**, **D**). (**A**) HPLC chromatogram of extracellular medium after incubation of cells with 100 *μ*M SN 23862 for 3 h at 2.5 × 10^5^ cells ml^−1^. U2, unknown product; ^*^phenol red. (**B**) Time course of formation of extracellular metabolites as in (**A**). (**C**, **D**) Appearance of metabolites in medium during incubation of SiHa-NTR^*puro*^ MCLs to SN 23862 (initial concentration 100 *μ*M in the donor compartment). Symbols as in (**B**).

SiHa-NTR^*puro*^ cells generated an SN 23862 extracellular metabolite profile (Figure 6A

) similar to V79-NTR^*puro*^ (Figure 5), with kinetics consistent with the expected precursor–product relationships (conversion of **9** to **10** and then **11**). When SN 23862 was reduced *in situ* in SiHa-NTR^*puro*^ MCLs, the appearance of metabolites in the medium (Figure 6C and D) showed a pattern similar to that in single-cell suspensions (Figure 6B). Concentrations of metabolites were lower on the receiver side as expected, but all the three diffused relatively efficiently. The initial flux of the bioactive metabolite **9** was ca 0.5 *μ*M h^−1^, which was faster than that of the less-potent CB 1954 metabolite **3** (initial flux ca 0.2 *μ*M h^−1^).

## DISCUSSION

An important feature of the NTR/CB 1954 enzyme/prodrug system for arming gene therapy vectors is that it generates cytotoxic product(s) capable of bystander killing of cells that do not express NTR. It has previously been shown that incubation of NTR-transduced cells with CB 1954 generates cytotoxic activity in extracellular medium, suggesting that the bystander effect results from passive diffusion of a lipophilic metabolite without any requirement for gap junctional communication or other cell–cell contact (Bridgewater *et al*, 1997). The latter study noted the formation of two extracellular radiolabelled metabolites suggested to be the 2- and 4-hydroxylamines. It has subsequently been assumed that the 4-hydroxylamine is the key mediator of CB 1954 bystander effects, because of its potent cytotoxicity in low cell density cultures. The present study challenges this view, and demonstrates that other reduction products from CB 1954, particularly its 2-amine **3** (CB 10-236), are likely to play important roles in its bystander effects in tumours.

We have used a high-resolution HPLC method, with on-line mass spectrometry, to identify five extracellular metabolites from CB 1954. Bioassay of the HPLC eluate against UV4 cells showed four of these (the 2- and 4-hydroxylamines **2** and **4**, and corresponding amines **3** and **5**) to have appreciable cytotoxicity. The amines have not previously been reported as end products of metabolism by NTR in cells, but their formation is expected, given the sensitivity of the hydroxylamines of CB 1954 to disproportionation to the nitroso and amino derivatives (Helsby *et al*, 2003). The loss of the 4-hydroxylamine (**4**) in culture medium, and the formation of the corresponding 4-amine (**5**) is little affected by the presence of cells (Figure 4), suggesting that spontaneous chemical conversion is the major pathway, but a contribution from cellular reduction of the hydroxylamines is not excluded at high cell density in tumours.

The biological importance of metabolites other than the 4-hydroxylamine is underscored by two observations. First, although **4** is more cytotoxic than the corresponding 2-hydroxylamine (**2**) against UV4 cells, it is not the most potent metabolite against repair-competent cells (Table 2). In particular, the 2-amine (**3**) has a lower IC_50_ than the 4-hydroxylamine (**4**) against the human tumour cell lines (SiHa and WiDr). The high cytotoxicity of the 2-amine (relative to the 4-amine or CB 1954) against AA8 cells has been reported previously (Kestell *et al*, 2000), and, in the original study on the formation of the 4-hydroxylamine (**4**) from CB 1954, it was also noted that the 2-amine is much more potent than the 4-amine against Walker cells (Knox *et al*, 1988b), although no direct comparison was made with the 4-hydroxylamine in either of these studies. The differential toxicity of the 2-amine (**3**) in the NER-defective line (ratio of AA8/UV4 IC_50_ values in Table 2) indicates that it is a DNA alkylator (like the 4-hydroxylamine, (**4**), but the lower ratio (7.1 *vs* 63) suggests that it may form monoadducts rather than crosslinks. The cytotoxicity of the 2-amine may be due to the electronic activation of the aziridine moiety, the nucleophilic reactivity of which is enhanced to a greater extent by reduction of the 2-NO_2_ than the 4-NO_2_ group of CB 1954 (Helsby *et al*, 2003). We have not excluded the possibility that **3** undergoes further bioactivation in target cells, such as by reduction of the 4-NO_2_ group by NTR or by another potential nitroreductase such as DT-diaphorase. Indeed, rat DT-diaphorase is known to metabolise **3** (Knox *et al*, 1988a). However, if any such secondary activation occurs, it does not result in a significant new bystander metabolite, as no unassigned bioactive peaks are observed in the extracellular medium.

The second observation is that the amine metabolites have greater diffusion ranges in 3D cell cultures than the corresponding hydroxylamines, as demonstrated by the change in metabolite profile in extracellular medium when CB 1954 is activated by SiHa-NTR^*puro*^ cells in MCLs *vs* single cells in suspension (Figure 3). In single-cell suspensions, the hydroxylamines are prominent, but when CB 1954 is reduced within MCLs the amines are the main species in the medium and obviously diffuse efficiently as they accumulate in the receiver compartment on the opposite side of the MCLs. The inefficient transport of the 4-hydroxylamine (**4**) relative to the corresponding amine (**5**) was confirmed by the failure of **4** to penetrate SiHa MCLs, while **5** (formed from **4** during this experiment) was able to do so (Figure 4). To the extent that MCLs model the extravascular compartment of tumours, this would indicate that the 4-hydroxylamine is unlikely to kill NTR−ve cells efficiently in tumours, unless they are immediately adjacent to the NTR+ve cells. The superior extravascular transport of the amines relative to the hydroxylamines is consistent with their slightly greater lipophilicity (lipophilicity substituent constant *π* −1.23 for NH_2_ *vs* −1.34 for NHOH; (Hansch and Leo, 1979) and considerably greater stability (Helsby *et al*, 2003 and this study).

When the yield, cytotoxicity and tissue-penetration properties are considered for each CB 1954 metabolite, the 2-amine (**3**) emerges as a critical extracellular metabolite and a leading contender as the species responsible for bystander effects of CB 1954 when activated by NTR in tumours. This is likely to be the dominant metabolite at longer diffusion distances, with the 4-hydroxylamine (**4**) possibly contributing to short-range bystander killing. The contribution of these species can be expected to change depending on the specific geometry of the diffusion problem, which, in turn, will reflect the microregional distribution of the NTR vector. We would predict, for example, that bystander effects for NTR-armed *Clostridia sporogenes* (Lemmon *et al*, 1997) localised in necrotic regions of tumours will depend to a greater extent on the 2-amine metabolite (**3**), while the 4-hydroxylamine (**4**) will make a larger contribution in tumours grown as intimate mixtures of NTR+ve and −ve cell lines. The formation of two distinct bystander metabolites might be an advantage if the mechanisms of cell killing by **3** and **4** differ to the extent that cross-resistance between them is incomplete.

These considerations call into question whether it would be advantageous to activate CB 1954 only by reduction at the 4-NO_2_ group. Anlezark *et al* (2002) have recently characterised such a reductase from *Bacillus amyloliquifaciens* and suggested that this may be preferable for GDEPT applications, because it provides only the more toxic 4-hydroxylamine metabolite. Our results suggest that reduction of the 2-NO_2_ group is at least as important. We would expect *E. coli* NTR to be preferable to rat DT diaphorase (which also reduces only the 4-NO_2_ group of CB 1954) for GDEPT application for this same reason, in addition to its faster kinetics of reduction (Knox *et al*, 1992).

Finally, this study shows that the nitrogen mustard analogue of CB 1954, SN 23862 (**7**), is reduced more rapidly than CB 1954 by NTR+ve cells (Table 1), as previously reported with purified NTR (Anlezark *et al*, 1995), and forms just a single bioactive extracellular metabolite when reduced in either V79-NTR^*puro*^ (Figure 5) or SiHa-NTR^*puro*^ cells (Figure 6). This metabolite is also the 2-amino reduction product (**9**), which has superior potency to any of the CB 1954 metabolites, especially against the two human cell lines tested (Table 2). The initial rate of appearance of this metabolite from SiHa-NTR^*puro*^ in extracellular medium (Figure 6B) is considerably faster than that of **3**+**4** from CB 1954 (Figure 3B), and it shows very efficient diffusion within SiHa-NTR^*puro*^ MCLs (Figure 6B, C). All these factors indicate that SN 23862 should provide more efficient bystander effects than CB 1954 in NTR-GDEPT; this is consistent with the reported superior bystander killing by SN 23862 in MCLs and xenografts comprising mixtures of NTR+ve and −ve WiDr cells (Wilson *et al*, 2002). Whether such metabolites might diffuse too well, and thus contribute to systemic toxicity, needs to be evaluated critically, but we have not seen evidence of increased toxicity to either CB 1954 or SN 23862 in mice bearing NTR-expressing tumours. Analogues of SN 23862 that provide more potent 2-amino metabolites, and which have improved formulation characteristics, are currently in development in this laboratory.
